# Multivariable Evaluation of Wireless Power Transfer in Electrified Pavements with Magnetite-Modified Asphalt Mixtures

**DOI:** 10.3390/s25216646

**Published:** 2025-10-30

**Authors:** Gustavo Boada-Parra, Federico Gulisano, Damaris Cubilla, Juan Gallego

**Affiliations:** 1 Departamento de Ingeniería del Transporte, Territorio y Urbanismo, Universidad Politécnica de Madrid, C/Profesor Aranguren 3, 28040 Madrid, Spain; federico.gulisano@upm.es (F.G.); damaris.cubilla@alumnos.upm.es (D.C.); juan.gallego@upm.es (J.G.); 2 College of Science and Engineering, Universidad San Francisco de Quito, Diego de Robles y Vía Interoceanica, Quito 170901, Ecuador; 3 Transport Research Centre—TRANSyT, Universidad Politécnica de Madrid, Calle Profesor Aranguren 3, 28040 Madrid, Spain

**Keywords:** wireless power transfer (WPT), inductive charging, magnetite-modified asphalt, asphalt mixtures, electrified pavements, magnetic filler, random forest analysis

## Abstract

**Highlights:**

**What are the main findings?**

- Magnetite increased received power up to 13% and efficiency gains up to 2%.
- Thicker asphalt layers reduced transfer, but magnetite mitigated these losses.
- Magnetite mixtures maintain WPT performance under thicker layers.

**What is the implication of the main finding?**

- Multivariate analysis guides optimal design of electrified pavements.

**Abstract:**

Electrified roads with embedded wireless power transfer (WPT) systems provide a promising strategy for dynamic charging of electric vehicles, but pavement materials strongly influence transmission efficiency. This study examines the effect of replacing conventional filler with magnetite powder in AC-16 asphalt mixtures. Specimens were prepared with five magnetite substitution levels (0–100%) and three bitumen contents (4.1%, 4.6%, and 5.1%) and were tested under different temperatures (10, 20, and 40 °C), moisture conditions (dry and saturated), and specimen thicknesses. Power transmission was measured with a resonant inductive system at 85 kHz, and both received power variation (RPV) and relative efficiency (RE) were computed. Results showed that magnetite systematically improved electromagnetic performance: RPV increased by up to 13% under dry conditions at 20 °C with 100% magnetite, while RE exhibited smaller variations (−1% to +2%). Moisture reduced RPV, and high temperature (40 °C) caused additional losses, whereas RE remained largely stable. Bitumen contributed indirectly, adding modest RPV gains. Thickness was the dominant geometric factor, with magnetite content particularly effective in mitigating losses at greater depths. Random forest analysis confirmed thickness and magnetite as the most influential variables. These findings demonstrate the potential of magnetite-modified asphalt to enhance the design of WPT-enabled pavements, providing a robust experimental basis for future full-scale applications.

## 1. Introduction

Transport accounts for approximately 8.2 Gt of CO_2_ in 2023 worldwide and nearly 25% of EU greenhouse gas emissions in Europe [1]. Road traffic is the primary contributor, with diesel-related CO_2_ and N_2_O emissions rising by 97% and 355% since 1990, while pollutants such as NO_2_ and PM continue to harm air quality and health [2,3]. The shift to electric vehicles (EVs) offers a direct way to cut tailpipe emissions. Global EV sales surpassed 17 million in 2024 (≈20% of total), raising the fleet to nearly 58 million units, with China leading at 11 million and emerging markets in Asia and Latin America growing by over 60% [4,5,6]. This rapid electrification demands advances in charging infrastructure, where wireless and dynamic charging are emerging as key solutions. Several international pilot projects—such as OLEV in South Korea, FABRIC in Europe, Smartroad Gotland in Sweden, Arena del Futuro in Italy, and Electreon trials in Israel and the USA—have already demonstrated the feasibility of Dynamic Wireless Power Transfer (DWPT) under real traffic conditions [7,8,9].

Advances in WPT technology have largely centered on optimizing coil configurations, such as the Double-D design proposed by Pahlavan et al. [10], which enhances magnetic coupling and efficiency at 85 kHz. However, limited attention has been given to how the pavement medium itself influences power transfer, despite its direct role in determining the effective magnetic path between coils. In electrified roads, the primary coil is embedded beneath the pavement and magnetically coupled to the secondary coil, which is mounted under the vehicle. The asphalt layer between them acts as the transmission medium for the magnetic field, reducing coupling efficiency. Energy transfer is governed not only by coil geometry and alignment but also by separation distance and the electromagnetic properties of the intervening material [11,12,13,14,15]. Conventional asphalt, with low magnetic permeability and insulating behavior, attenuates the field and weakens coil coupling, becoming a significant limitation for efficient WPT in pavements [16].

Pavement properties such as dielectric constant, moisture content, air voids, and thickness further influence electromagnetic transmission. Chen et al. [17] reported dielectric losses in asphalt and concrete and showed that concrete resistivity affects WPT more than permittivity. Li et al. modeled energy losses considering dielectric properties and pore water [18]; Zhai et al. linked aggregate size to asphalt dielectric response [19]; and Abufares & Al-Qadi developed predictive protocols based on aggregate dielectric constants [20]. These factors are critical in dynamic systems, where small losses can significantly reduce power transfer. To address them, conductive and ferromagnetic additives have been studied: ferrite powders to increase permeability [21,22], steel fibers for induction heating and magnetic sensing [23,24], fly ash to improve stiffness and fatigue resistance [25], and magnetite to enhance coupling and efficiency in asphalt mixtures [26,27,28,29,30].

In recent years, researchers have improved the magnetic response of pavement materials by incorporating ferromagnetic additives, such as ferrites, iron filings, and magnetite (Fe_3_O_4_). Magnetite is particularly promising due to its high magnetic permeability and compatibility with asphalt. Magnetite has been preferred over other ferromagnetic fillers such as ferrite or steel fibers because it offers a balanced combination of electromagnetic performance, stability, and cost efficiency. It exhibits moderate magnetic permeability ($\mu_{r}$ ≈ 10–150) and low electrical conductivity, which minimizes eddy-current losses during WPT. Although ferrites can achieve much higher permeability ($\mu_{r}$ up to 10^4^), their high-temperature sintering process and cost limit their large-scale use in pavements. Steel fibers ($\mu_{r}$ ≈ 500–900) often lead to agglomeration, increased air voids, and local distortion of magnetic fields. In contrast, magnetite is a natural, low-cost mineral that disperses uniformly within bitumen, providing an optimal balance between coupling efficiency and workability [21,22,24,26,31,32,33]. Experimental and numerical studies show that its inclusion increases mutual inductance and coupling efficiency by directing magnetic flux through the pavement layer. However, excessive content may induce magnetic losses, eddy currents, or shielding effects that offset these gains [16,26]. Optimizing dosage and distribution to achieve magnetic gains without compromising material integrity is also essential.

Despite these advances, few studies have systematically examined the combined effects of magnetite content with bitumen proportion, moisture, temperature, or layer thickness on WPT efficiency [16,18,26,31,34]. Previous research has usually addressed electromagnetic or mechanical behavior separately, while the literature also indicates that magnetite can influence durability. This work focuses on the electromagnetic response, isolating and quantifying the electromagnetic impact of magnetite-modified asphalt mixtures under controlled laboratory conditions.

Using AC-16 dense-graded asphalt, specimens were prepared with varying magnetite filler levels (0–100%), bitumen contents, moisture states, temperatures (10, 20, and 40 °C), and pavement thicknesses. WPT performance was measured with a resonant coil system at 85 kHz, and a random forest algorithm was applied to identify the relative influence of each factor and its interactions.

The results show that magnetite significantly improves WPT efficiency, confirming its potential for electrified roads. These findings provide a scientific basis for optimizing asphalt mixture design for electromagnetic performance and lay the groundwork for future studies on mechanical durability and field implementation.

## 2. Materials and Methods

### 2.1. Asphalt Mix Design

An AC-16 dense-graded asphalt mixture was used as the base material, prepared with crushed porphyry aggregates and a 50/70 penetration-grade bitumen, in compliance with European (UNE) standards and PG-3 Spanish specifications. Substitution was performed on a volumetric basis, considering the specific gravities of CaCO_3_ (2.7 g/cm^3^) and Fe_3_O_4_ (4.6 g/cm^3^). The synthetic magnetite powder used (>96.8% Fe_3_O_4_) consisted of spherical particles (<45 µm, predominant size ≈ 200 nm) with a bulk density of 0.8–1.2 g/cm^3^ and a true density of 4.6 g/cm^3^.

**Figure 1:**
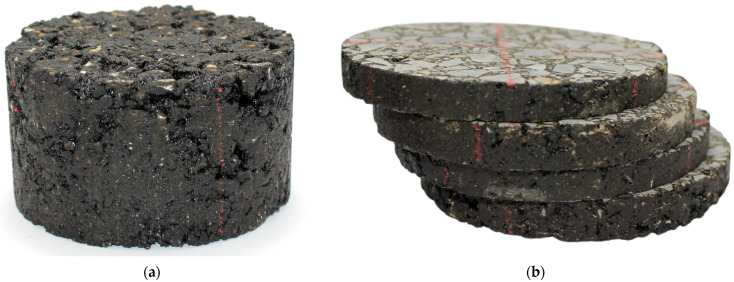
Materials used in WPT tests: (**a**) Marshall specimen of AC-16 asphalt mixture; (**b**) disks sectioned from the Marshall specimen for wireless power transfer measurements.

Three bitumen contents—4.1%, 4.6%, and 5.1% by total mixture weight—were selected to investigate their influence on electromagnetic performance. Marshall-compacted cylindrical specimens were fabricated for each magnetite–bitumen combination (5 substitution percentages × 3 bitumen contents × 4 replicates = 60 Marshall specimens). Each specimen was subsequently sectioned into four disks intended for WPT testing. The resulting mixtures showed apparent densities between 2.41 and 2.47 g/cm^3^ and air void contents ranging from 5.96% to 2.17%, decreasing with increasing bitumen and magnetite contents, indicating improved compaction and reduced porosity. The resulting specimens exhibited thicknesses ranging from 7.26 mm to 14.54 mm (mean = 10.39 mm, SD = 0.96 mm) (Figure 1b). These disks were used for WPT testing under different thicknesses and environmental conditions (dry and saturated surface-dry). The complete testing program is described in Section 2.4.1, comprising a total of 1800 individual tests.

### 2.2. Pre-Test Conditioning Protocol

Specimens were conditioned under controlled thermal and moisture conditions to assess their influence on WPT performance. Two conditioning states were considered: dry and saturated surface-dry (SSD).

**Figure 2:**
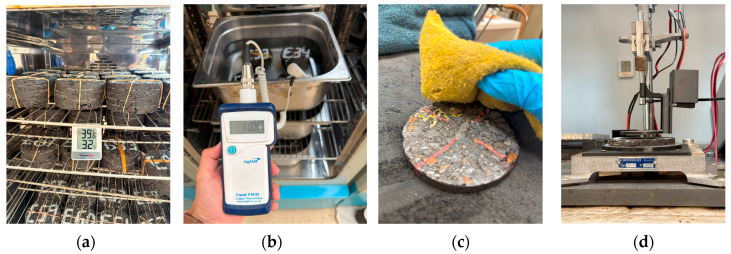
Environmental conditioning protocol: (**a**) dry temperature chamber; (**b**) SSD conditioning in water; (**c**) surface drying; (**d**) disk transfer to WPT setup.

In the dry condition, disks were stored at three target temperatures (10 °C, 20 °C, and 40 °C) inside a temperature-controlled chamber for at least 24 h (Figure 2a). Immediately prior to testing, each disk was removed and transferred to the WPT setup, which had been pre-stabilized at the same temperature as the test (Figure 2d).

In the SSD condition, disks were submerged in water containers placed inside the temperature chamber for 24 h at 10 °C, 20 °C, and 40 °C (Figure 2b). Before measurement, each disk was removed, surface-dried with a cloth to remove excess water (Figure 2c), and immediately transferred to the WPT setup, which was pre-stabilized at the target temperature. Water absorption ranged from 2.17% to 5.84% (mean = 4.04%), decreasing with increasing bitumen and magnetite contents. This confirms that the SSD condition represents partial saturation, mainly affecting interconnected pores.

### 2.3. WPT Measurement Setup

**Figure 3:**
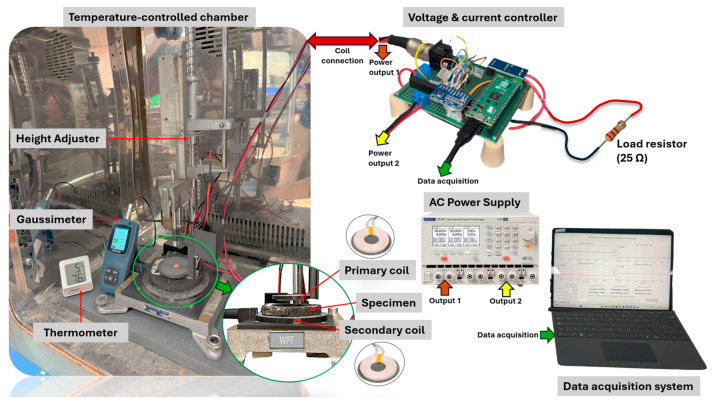
Wireless Power Transfer (WPT) measurement setup with primary (transmitting) and secondary (receiving) coils aligned, and an asphalt mixture disk placed between them (1.5 cm separation).

The electromagnetic response of the AC16 mixtures was evaluated using a resonant inductive wireless power transfer (WPT) system (Figure 3). The setup comprised primary (transmitting) and secondary (receiving) circular coils, each with eight turns, a 2.0 cm central opening, and an outer diameter of 4.8 cm. The coils (designed initially as commercial low-power units for electronic devices) operated at 85 kHz. Both coils were placed inside a temperature-controlled chamber and allowed to stabilize for at least one hour at the target temperature. At the same time, the voltage source, controller, and data acquisition system remained outside to prevent thermal interference. A fixed vertical separation of 1.5 cm was set. The primary coil was powered by a voltage-limited source (≤30 V), while the secondary coil was connected to a 25 Ω resistive load through a voltage–current controller.

Asphalt mixture disks, sectioned from Marshall specimens, were placed between the coils inside a temperature-controlled chamber to ensure consistency with the pre-conditioning protocol (Section 2.2). Before each measurement, a 30 s air-gap reading was performed with no specimen between the coils to establish the reference condition. During this stage, the instantaneous transmitted and received powers, $P_{t,air\text{-}gap}(t)$ and $P_{r,air\text{-}gap}\left(t\right)\;$ [W], were continuously recorded at approximately 2 Hz to obtain the baseline signals used for later relative comparisons.

Subsequently, the asphalt mixture disks were inserted between the coils, and each test was conducted under identical acquisition conditions. A 5 s stabilization period preceded the 30 s of continuous data recording at the same sampling frequency (≈2 Hz). During this step, the instantaneous transmitted and received powers, $P_{t}(t)$ and $P_{r}\left(t\right)$ [W], were recorded for each specimen. The instantaneous Power Transfer Efficiency, *PTE*(*t*) [%] and *PTE_air-gap_*(*t*) [%] were calculated at each sampling point as:

(1) $$PTE\left(t\right)=\frac{P_{r}\left(t\right)}{P_{t}\;\left(t\right)}\times 100;\;\;\;{PTE}_{air\text{-}gap}(t)=\frac{P_{r,air\text{-}gap}(t)}{P_{t,air\text{-}gap}\;(t)}\times 100$$

### 2.4. Statistical Analysis

#### 2.4.1. Data Preparation

The continuous signals recorded during both air-gap and asphalt mixture specimen measurements were post-processed to obtain representative mean values of transmitted and received power, averaged over the corresponding acquisition windows, as:

(2) $$\bar{P_{t}}=\frac{1}{T}\int_{0}^{T}P_{t}\left(t\right)dt;\;\;\;\bar{P_{r}}=\frac{1}{T}\int_{0}^{T}P_{r}\left(t\right)dt$$

where *T* = 30 s for the asphalt mixture specimen measurements and for the air-gap reference tests. The same averaging procedure was applied to the reference signals to obtain $\bar{P_{t,air\text{-}gap}}$ and $\bar{P_{r,air\text{-}gap}}\;$ [W].

From the instantaneous Power Transfer Efficiency defined in Equation (1), *PTE*(*t*), which was recorded as a continuous signal during each test, its mean value over the acquisition window was calculated analogously for both the air-gap and asphalt mixture measurements, as:

(3) $$\bar{{PTE}_{air\text{-}gap}}=\frac{1}{T}\int_{0}^{T}{PTE}_{air\text{-}gap}\left(t\right)dt;\;\;\;\bar{PTE}=\frac{1}{T}\int_{0}^{T}PTE\left(t\right)dt;$$

The Received Power Variation (RPV) [W] and the Relative Efficiency (RE) [pp] were obtained as the differences between the mean values of the specimen and the air-gap measurements for $\bar{P_{r}}$ and $\bar{PTE}$, respectively:

(4) $$RPV=\bar{P_{r}}-\bar{P_{r,air\text{-}gap}}$$

(5) $$RE=\bar{PTE}-\bar{{PTE}_{air\text{-}gap}}$$

The processed records were compiled in Python 3.10 (using the pandas library) into a dataset for statistical and machine learning analyses. Each disk contributed one record. The dataset contained 1800 entries and seven variables: five independent—bitumen content (4.1, 4.6, 5.1%), magnetite substitution (0, 25, 50, 75, 100%), temperature (10, 20, 40 °C), moisture state (dry, SSS), and specimen (disk) height—and two dependent—RPV and RE. Thickness was measured individually and incorporated as a continuous predictor in the multivariable regression and Random Forest models. Outliers identified through frequency-distribution screening (RPV < −40 W or RE < −10 pp), which were attributed to procedural issues, were removed (n = 8; <0.5%), resulting in 1792 valid observations. The cleaned dataset was split using scikit-learn into training (80%) and testing (20%) subsets to evaluate predictive performance on unseen data and reduce overfitting.

#### 2.4.2. Methodology for Multiple Linear Regression Models

Multiple linear regression (MLR) assumes that the dependent variable is a linear combination of the predictors plus an error term.

(6) $$Y=\beta_{0}+\beta_{0}X_{0}+\beta_{2}X_{2}+\ldots +\beta_{n}X_{n}+\varepsilon$$

where *Y* is the dependent variable (RPV or RE), *β*_0_ is the intercept, *β*_*i*_ are the regression coefficients, *X*_*i*_ are the independent variables (bitumen content, magnetite content, temperature, moisture state, specimen height), and *ε* is the random error.

Model parameters were estimated by least squares, minimizing the sum of squared residuals between observed and predicted values. Model performance was evaluated using the coefficient of determination (*R*^2^):

(7) $$R^{2}=\frac{\sum {\left(y_{i}-{\hat{y}}_{i}\right)}^{2}}{\sum {\left(y_{i}-{\bar{y}}_{i}\right)}^{2}}$$

where $y_{i}$ denotes the observed values, ${\hat{y}}_{i}$ the predicted values, and ${\bar{y}}_{i}$ the mean of observed values.

#### 2.4.3. Methodology for Random Forest Models

Random Forest (RF) is an ensemble learning algorithm that constructs multiple decision trees and aggregates their predictions, improving robustness and accuracy over single trees [35]. The random forest algorithm has found several applications in pavement engineering, such as estimating the International Roughness Index (IRI) [35], assessing aggregate properties that affect asphalt pavement friction [36], or predicting mechanical properties in cold recycled asphalt mixtures [36].

Unlike linear regression, which assumes linear relationships between predictors and responses, RF can capture complex non-linear patterns, feature interactions, and threshold effects without requiring explicit specification. Given the complexity of the electromagnetic phenomena analyzed in this study, RF regression models were developed to predict RPV and RE using the same independent variables considered in the linear regression models.

Two key hyperparameters were tuned:

- Number of estimators (n_estimators): 10, 100, 200, and 300.
- Maximum depth (max_depth): 3, 5, 10, and 15.

Model optimization employed 5-fold cross-validation. The training dataset was randomly partitioned into five subsets; in each iteration, four folds were used for training and one for validation. The mean validation score across folds was used to identify the best hyperparameter combination, ensuring robust performance estimates and reducing sensitivity to data partitioning. The test set remained completely unseen during hyperparameter optimization and was used exclusively after model training to validate predictive performance.

Final models with optimized parameters were trained separately for RPV and RE. Model performance was assessed using the coefficient of determination (*R*^2^), computed with the same formulation for both multiple linear regression and Random Forest models to ensure consistency in comparison (Equation (7)). Model interpretability was further analyzed using SHapley Additive exPlanations (SHAP), which quantify the marginal contribution of each predictor to the model outputs.

Finally, the optimized RF models were used to generate three-dimensional response surfaces illustrating the combined effects of bitumen and magnetite contents on RPV and RE. Other variables were held at reference values (mean conditions), enabling visualization of the design space and identification of optimal mixture configurations.

## 3. Results

### 3.1. Multiple Linear Regression Models

The results of the multiple linear regression analysis are summarized in Equations (8) and (9), which present the fitted prediction equations for RPV and RE, respectively:

(8) RPV=8.1451−1.0991·Height+0.6568·Bitumen+0.1249·Magnetite−0.0525·Temperature+0.4480·Condition_Dry

(9) RE=1.5515−0.2711·Height+0.2469·Bitumen+0.0228·Magnetite−0.0066·Temperature−0.0313·Condition_Dry

For the RPV model (Equation (8)), specimen height shows the strongest influence among the predictors, with a negative coefficient (−1.0991) indicating that increasing thickness attenuates magnetic field coupling and reduces received power. Bitumen (0.6568) and magnetite (0.1249) contents have a positive effect, enhancing electromagnetic transmission. The influence of temperature is minor (−0.0525), and the binary variable *Condition_Dry* (0 = SSD; 1 = Dry) confirms that dry specimens yield higher received power.

For the RE model (Equation (9)), the same qualitative trends are observed, although the overall sensitivity of RE to the predictors is smaller due to its limited range of variation. Within this model, height again shows the largest (negative) effect, while bitumen and magnetite contribute positively. Temperature and moisture conditions remain nearly neutral, indicating that RE is comparatively less affected by these variables than RPV.

**Figure 4:**
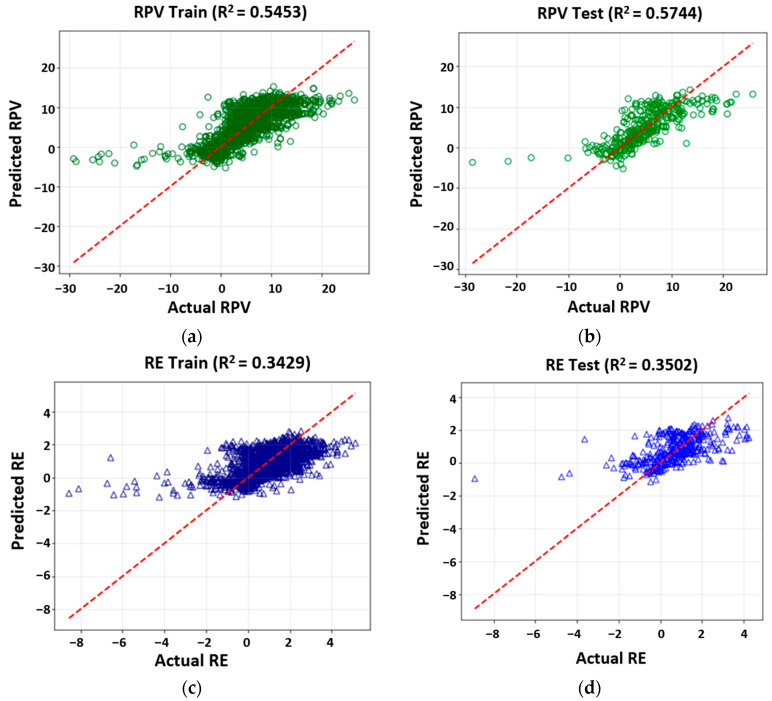
Observed versus predicted values from multiple linear regression (MLR) models for RPV and RE. (**a**) RPV—Training set; (**b**) RPV—Testing set; (**c**) RE—Training set; (**d**) RE—Testing set.

Figure 4 compares observed and predicted values for the training and test datasets. The RPV model shows moderate predictive performance, with *R*^2^ values of 0.5453 (training, Figure 4a) and 0.5744 (testing, Figure 4b), indicating that approximately 55% of the variance is explained without evidence of overfitting. In contrast, the RE model presents substantially lower predictive capability, with *R*^2^ values of 0.3429 (training, Figure 4c) and 0.3502 (testing, Figure 4d), explaining only about 34–35% of the variance.

These results highlight that (i) linear regression can partially capture the influence of mix design and environmental parameters on electromagnetic behavior, particularly for transmitted power, and (ii) the relatively low *R*^2^ values, especially for RE, indicate that power transfer phenomena in magnetite-modified asphalt are governed by non-linear interactions not adequately represented by linear models. This limitation suggests that either additional predictor variables are required or that the underlying physical processes are inherently non-linear. Accordingly, non-linear approaches such as Random Forest were explored to improve predictive accuracy and capture the complexity of electromagnetic interactions.

### 3.2. Random Forest Models

**Figure 5:**
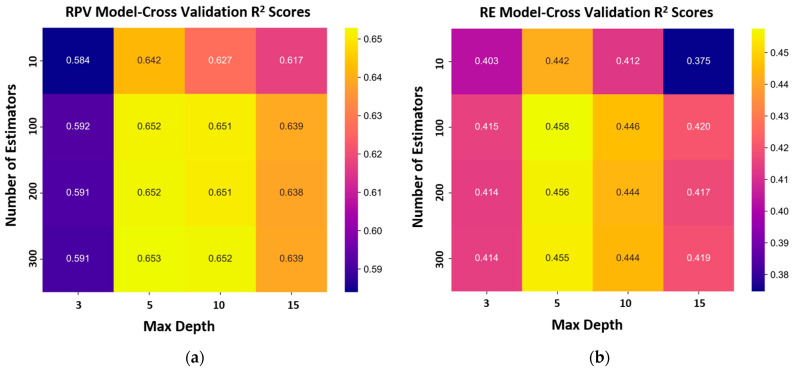
5-fold cross-validation results for random forest models: (**a**) RPV and (**b**) RE.

The results of the k-fold cross-validation procedure for hyperparameter tuning are presented in Figure 5. The grid search systematically evaluated different combinations of the number of estimators and maximum tree depth to identify the optimal model configurations for RPV and RE predictions. For the RPV model (Figure 5a), the optimal configuration was achieved with 300 estimators and a maximum tree depth of 5, resulting in a 5-fold cross-validation *R*^2^ of 0.653. In contrast, the RE model (Figure 5b) achieved optimal performance with 100 estimators and a maximum tree depth of 5, yielding a 5-fold cross-validated *R*^2^ of 0.458. This indicates that fewer trees were sufficient to maximize predictive accuracy. That moderate tree depth provided the best balance between bias and variance, avoiding overfitting while maintaining stable generalization performance.

**Figure 6:**
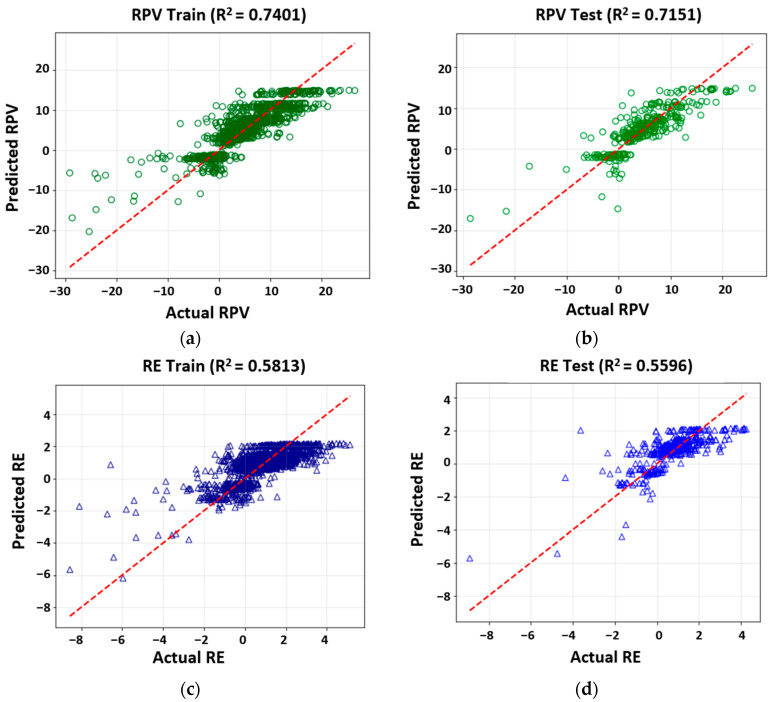
Observed versus predicted values from random forest (RF) models for RPV and RE. (**a**) RPV—Training set; (**b**) RPV—Testing set; (**c**) RE—Training set; (**d**) RE—Testing set.

The optimized hyperparameters were used to develop the final Random Forest models, with results shown in Figure 6. It is important to note that the *R*^2^ values obtained from k-fold cross-validation differ from those of the final models. This difference is expected and arises from the inherent nature of cross-validation. During hyperparameter optimization, the reported *R*^2^ represents the average performance across k iterations, where each iteration trains on a subset of the data (k-1 folds) and validates on the remaining fold. In contrast, the final model is trained using the optimized hyperparameters on the entire training dataset, resulting in a different *R*^2^ value. Substantial improvements over multiple linear regression were observed. For RPV, the test set *R*^2^ increased from 0.5744 to 0.7151 (+24.5% explained variance). The final model achieved *R*^2^ values of 0.7401 for training (Figure 6a) and 0.7151 for testing (Figure 6b), confirming good generalization with minimal overfitting. Similarly, the RE model improved from a baseline test *R*^2^ of 0.3502 to 0.5596 (+59.8%). Training and test *R*^2^ values were 0.5813 (Figure 6c) and 0.5596 (Figure 6d), respectively, again indicating consistent generalization. These results confirm the superior capability of Random Forest in capturing the non-linear relationships governing electromagnetic power transfer in magnetite-modified asphalt mixtures.

**Figure 7:**
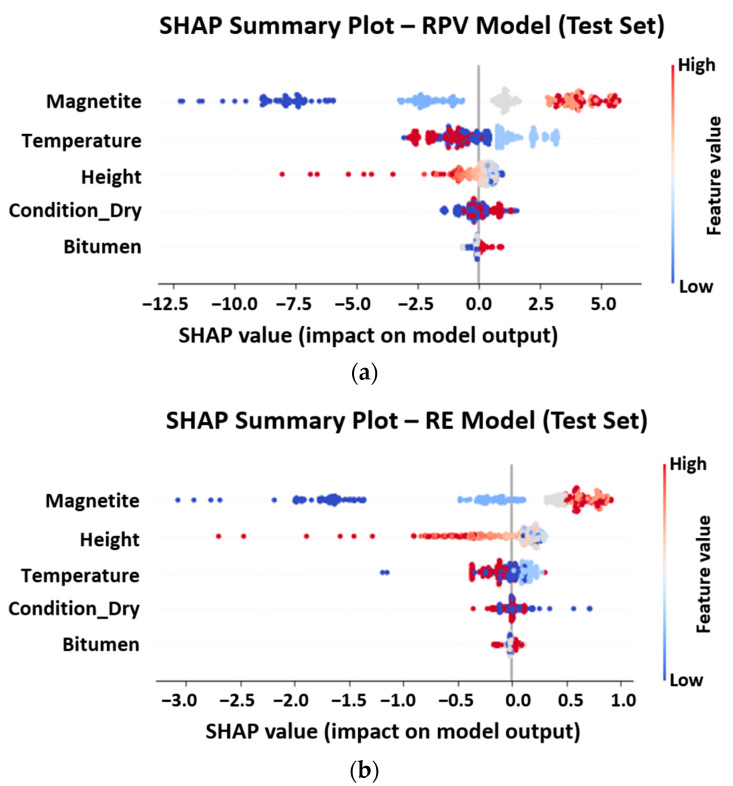
SHAP summary plots showing variable importance for RPV (**a**) and RE (**b**).

To interpret the influence of variables, SHAP analysis was performed, with the results shown in Figure 7a,b. The SHAP summary plots demonstrate that magnetite content is the most influential factor for both RPV and RE, with higher concentrations consistently improving electromagnetic performance (increased RPV and RE). Bitumen content exerts a moderate positive effect on RPV but only marginally influences RE. Temperature shows a non-monotonic trend, where both low and high extremes reduce performance, while intermediate values are favorable. Moisture condition exhibits contrasting effects: dry specimens yield higher RPV, whereas saturated-surface-dry (SSD) specimens slightly enhance RE. Finally, specimen height exerts a consistent negative impact on both metrics, confirming the attenuating effect on the electromagnetic coupling of greater material thickness.

**Figure 8:**
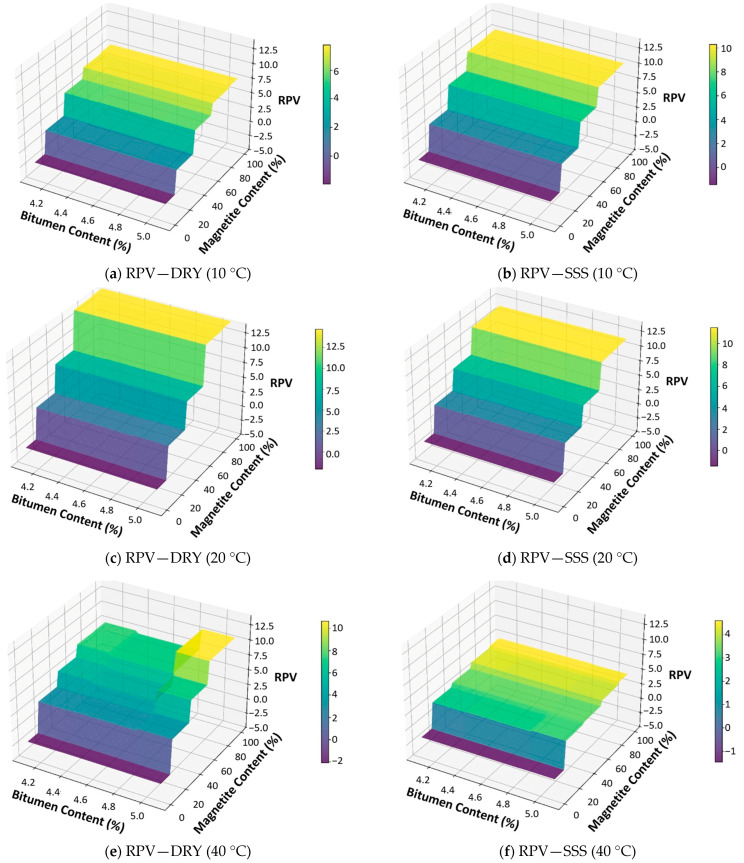
Random forest response surfaces for RPV showing the combined influence of bitumen and magnetite contents under dry (**a**,**c**,**e**) and SSD (**b**,**d**,**f**) conditions at 10 °C, 20 °C, and 40 °C.

**Figure 9:**
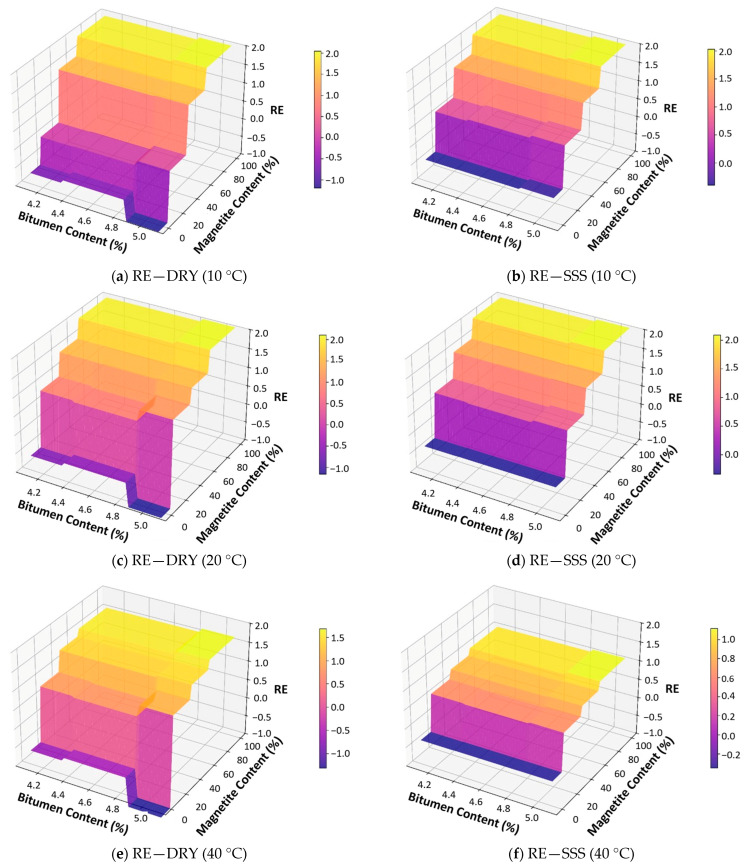
Random forest response surfaces for RE showing the combined influence of bitumen and magnetite contents under dry (**a**,**c**,**e**) and SSD (**b**,**d**,**f**) conditions at 10 °C, 20 °C, and 40 °C.

The Random Forest outputs were further exploited to construct 3D response surfaces (Figure 8 and Figure 9), enabling mix design optimization for magnetite-modified asphalt under different environmental conditions. These plots illustrate the interaction between bitumen and magnetite contents on electromagnetic performance, with specimen height fixed at its average value. The stepped appearance of the surfaces reflects the discrete values tested in this study, suggesting that future research should include additional intermediate levels and extended ranges to refine optimization.

The comparison between RPV and RE highlights differences in magnitude and sensitivity. RPV achieves gains up to +12% to 13% under dry conditions at 20 °C with 100% magnetite and optimal bitumen (4.6–5.1%), whereas RE exhibits a narrower range of −1% to +2%, peaking at +2% in the same scenario. Moisture systematically reduces RPV (−1 to −3 pp), especially at 40 °C, but for RE it does not penalize performance and in some cases slightly enhances it (+0.2–0.3 pp). Temperature has a stronger effect on RPV, with decreases of 2–5 pp at 40 °C, while RE remains largely unaffected. Overall, magnetite is critical for both metrics, though its effect is more pronounced on received power than on efficiency, with RE being less sensitive to environmental conditions.

## 4. Discussion

The results demonstrate that magnetite influences both received power variation (RPV) and relative efficiency (RE) in asphalt mixtures, with apparent differences in magnitude. RPV rose by up to 13% under dry conditions at 20 °C with 100% magnetite and 4.6–5.1% bitumen. RE remained within a narrower range of −1% to +2%, reaching its maximum under the same conditions. This shows that magnetite has a more substantial impact on coupled power than on overall efficiency. The behavior of the resonant circuit can explain the phenomenon: the higher magnetic permeability of magnetite increases mutual inductance and thus the received power ($\bar{P_{r}}$), but the system simultaneously compensates by slightly raising the transmitted power ($\bar{P_{t}}$) to maintain resonance. As both $\bar{P_{r}}$ and $\bar{P_{t}}$ rise proportionally, their ratio ($\frac{\bar{P_{r}}}{\bar{P_{t}}}$) remains nearly constant, resulting in limited changes in RE.

Magnetite substitution also affected the mixture’s volumetric characteristics. Replacing CaCO_3_ (2.7 g/cm^3^) with Fe_3_O_4_ (4.6 g/cm^3^) increased apparent density from 2.41 to 2.47 g/cm^3^ and reduced air voids from 5.96% to 5.14% at 4.1% bitumen, and from 2.77% to 2.17% at 5.1%. This densification may reduce dielectric losses and moisture ingress, improving both electromagnetic coupling and mechanical durability.

The obtained values align with previous studies. Cui et al. [26] reported a 36% increase in output power with 10% magnetite. Guo and Wang [16] observed efficiency gains of 1.5–12.3% in FEM simulations with partially magnetized layers, consistent with the limited RE improvements found here. In contrast, Mahmud et al. [30] measured an efficiency increase from 13% to 86% in cementitious media with magnetic nanoparticles, which is not directly comparable to bituminous mixtures.

The present work simultaneously evaluated five variables: magnetite content, bitumen content, temperature, moisture, and specimen thickness. Previous studies focused on one or two factors: Cui et al. [26] on magnetite and thickness, Rizelioglu et al. [31] on bitumen, and Li et al. [21] and Yu et al. [28] on dielectric properties. None included moisture or temperature in controlled tests. The present approach quantifies combined effects that were previously unaddressed.

Moisture reduced RPV by 1 to 3 percentage points (pp), consistent with Chen et al. [17], who linked pore water to dielectric losses. However, RE remained within the same range, indicating that system efficiency did not degrade under wet conditions. Temperature at 40 °C decreased RPV by 2–5 pp compared to 20 °C, while RE remained unchanged. This behavior likely results from competing dielectric mechanisms within the asphalt matrix: at low temperature (~10 °C), reduced molecular mobility limits dipolar polarization, weakening magnetic coupling, whereas at high temperature (~40 °C), increased electrical conductivity and dielectric losses (tan δ) detune the resonance and reduce transfer efficiency. Around 20 °C, polarization remains active while losses are moderate, producing the observed optimum. Because magnetite’s permeability is nearly temperature-independent, the response is primarily governed by the dielectric behavior of the bituminous matrix. These environmental effects on electromagnetic performance had not been experimentally validated in earlier studies.

Overall, the results confirm that the performance of electrified pavements depends on the combined influence of magnetite, bitumen, and environmental conditions. This integrated approach expands the available experimental evidence and provides design parameters applicable to the development of dynamic wireless power transfer (DWPT) road systems.

## 5. Conclusions

The inclusion of magnetite in asphalt mixtures enhanced wireless power transfer performance. Received power variation (RPV) increased by up to 13% under dry conditions at 20 °C with 100% magnetite and 4.6–5.1% bitumen, while relative efficiency (RE) showed smaller but consistent changes in the range of −1% to +2%.

Environmental factors influenced RPV more than RE. Moisture reduced received power by 1 to 3 pp, and high temperature (40 °C) decreased it by 2–5 percentage points (pp) compared to 20 °C. In contrast, RE remained stable, indicating that efficiency is less sensitive to environmental variations.

Bitumen content and specimen thickness also played a role. Higher bitumen levels contributed an additional +1 to 3 pp to RPV, whereas greater disk thickness consistently reduced both RPV and RE, attenuating the effect of magnetic coupling between coils.

This study advances previous research by simultaneously evaluating magnetite content, bitumen content, temperature, moisture, and thickness, providing a comprehensive basis for mixture optimization in electrified pavement applications. Although the experimental coils used were not designed explicitly for large-scale pavement systems, the relative comparisons remain robust. Future work will first extend this research under controlled laboratory conditions to dynamic operation, including coil misalignment and vehicle motion, supported by finite element electromagnetic simulations to assess scalability. Subsequently, optimized coil designs and full-scale trials will be developed to validate these findings under real traffic and environmental conditions.

## Data Availability

The data that support the findings of this study are available from the corresponding author upon reasonable request.

## Abbreviations

The following abbreviations are used in this manuscript:

AC16	Asphalt Concrete 16 mm
CaCO_3_	Calcium Carbonate
CU	Charging Unit
EV	Electric Vehicle
DWPT	Dynamic Wireless Power Transfer
Fe_3_O_4_	Magnetite (Iron(II,III) Oxide)
RPV	Received Power Variation
RE	Relative Efficiency
PTE	Power Transfer Efficiency
SSD	Saturated Surface-Dry
MLR	Multiple Linear Regression
RF	Random Forest
SHAP	SHapley Additive exPlanations
PG-3	Pliego de Prescripciones Técnicas Generales para Obras de Carreteras y Puentes (Spanish pavement specification)
WPT	Wireless Power Transfer
